# Implementing and Evaluating a Mobile Phone–Supported and Family-Centered Rehabilitation Program for People With Stroke in Uganda (F@ce 2.0): Protocol for a Randomized Controlled Trial

**DOI:** 10.2196/60955

**Published:** 2024-09-25

**Authors:** Gunilla Eriksson, Julius Tunga Kamwesiga, Uno Fors, Tonny Oyana, Lena von Koch, Charlotte Ytterberg, Susanne Guidetti

**Affiliations:** 1 Department of Neurobiology, Care Sciences and Society Karolinska Institutet Stockholm Sweden; 2 Uganda Allied Health Examinations Board Kampala Uganda; 3 Department of Computer and Systems Sciences Stockholm University Stockholm Sweden; 4 College of Computing and Information Sciences Makerere University Kampala Uganda; 5 Women’s Health and Allied Health Professionals Theme Karolinska University Hospital Stockholm Sweden

**Keywords:** information and communication technology, ICT, mobile health, mHealth, telehealth, telemedicine, remote rehabilitation, activity of daily living, ADL, East Africa, sub-Saharan Africa, intervention, occupational therapy, physiotherapy

## Abstract

**Background:**

Stroke is a global societal challenge. Annually, 13 million people experience stroke, and the prevalence of stroke is increasing in low-income countries; hence, accessible rehabilitation needs to be developed. Information and communication technology can help by providing access to rehabilitation support through information, self-evaluation, and self-management of rehabilitation. The F@ce 2.0 rehabilitation program provides support in goal-setting and problem-solving strategies through phone calls from the interventionist twice a week and daily SMS text message reminders over 8 weeks to improve performance in valued activities in everyday life. Our hypothesis is that F@ce 2.0 will increase functioning in daily activities and participation in everyday life as well as improve performance and satisfaction in valued daily activities and self-efficacy (ie, confidence in own ability to perform activities) among people living with the consequences of stroke.

**Objective:**

This study aims to implement F@ce 2.0, a mobile phone–supported and family-centered rehabilitation program, and evaluate its effects on performance in daily activities and participation in everyday life in comparison to ordinary rehabilitation among persons with stroke and their family members in Uganda. An additional aim is to explore experiences of participating in F@ce 2.0 and plausible mechanisms of impact that might explain the potential effects of F@ce 2.0.

**Methods:**

A randomized controlled trial will be conducted to compare the outcomes of the F@ce 2.0 group and a control group receiving ordinary rehabilitation. Health care professionals will recruit 90 clients from both urban and rural areas. The primary outcomes for persons with stroke are perceived performance in daily activities assessed using the Canadian Occupational Performance Measure and self-efficacy assessed using the Self-Efficacy Scale; for family members, the primary outcome is caregiver burden evaluated using the Caregiver Burden Scale. Descriptive statistics will be used to present characteristics and outcomes at 3 and 6 months. All statistical analyses comparing the outcomes at the different time points between the F@ce 2.0 and control groups will be performed using intention-to-treat analysis. Qualitative interviews will be used to explore the experiences of persons with stroke and their family members participating in F@ce 2.0, using a grounded theory approach to data collection and analysis. A process evaluation will be conducted using a single-case study design with mixed methods to explore the implementation process.

**Results:**

Recruitment and data collection in the randomized controlled trial were initiated in January 2022 and have been completed. The intervention has been provided to 51 participants in the intervention group. Interviews of persons with stroke, family members, and health care professionals have been conducted. Data analysis will be performed during autumn 2024 and spring 2025.

**Conclusions:**

This study will provide evidence of the plausible effects of F@ce 2.0 and the process of implementing the program in low-income countries.

**International Registered Report Identifier (IRRID):**

DERR1-10.2196/60955

## Introduction

### Background

The point of departure for this study is that all people living with a disability and their families have needs and the right to participate and engage in everyday life. Social participation [1] and engagement in daily activities [2] are strongly associated with health and well-being [3]. Everyday life changes often occur for people with stroke and their families [4], and there is a need for rehabilitation and support to enable functioning in daily activities and social participation in the community.

Stroke is a noncommunicable disease and a global societal challenge according to the World Health Organization [5]. Annually, >13 million people experience stroke, which means that worldwide 1 in 4 people aged >25 years will have stroke in their lifetime, and one-third will subsequently live with remaining disabilities [6]. The burden of stroke is increasing substantially in Africa [7,8], which has some of the highest incidence rates in the world [9]. Stroke currently ranks as the sixth leading cause of mortality in Uganda [10].

Empirical studies on the effectiveness of rehabilitation after stroke have shown positive effects of activities of daily living (ADLs) interventions [11]. However, most research and evidence for beneficial rehabilitation interventions after stroke originates from high-income countries, and evidence is lacking that such interventions can be implemented with similar outcomes in the context of sub-Saharan Africa [8]. In 2021 in Uganda, 41% of the population was living below the poverty line, subsisting on the equivalent of US $1.90 per day [12]. Furthermore, the majority of the Ugandan population (74%) lived in rural areas, where medical rehabilitation was almost nonexistent [13]. In addition to the poor socioeconomic conditions in Uganda, access to rehabilitation services can be limited due to poor infrastructure, inadequate numbers of rehabilitation professionals, and poor health support systems.

To increase accessibility to rehabilitation services, the development and implementation of contextually appropriate rehabilitation programs is urgently needed. One way to increase accessibility to rehabilitation could be to use the knowledge of health care professionals (HCPs) to train village health workers, who in turn can provide rehabilitation support in agreement with the model for community-based rehabilitation (CBR) [14]. The CBR model respects diversity and aims to empower people with disabilities and reduce stigma against them; it is also in line with a client- and family-centered approach.

In addition to causing disabilities, stroke can lead to a stressful situation for family members, with risk for depression, perceived caregiver burden [15], social isolation, physical problems, and decreased life satisfaction [16]. In Uganda, families are commonly involved after stroke and take responsibility for care not only in the acute hospital setting but also in community living [17]. Rehabilitation programs after stroke should therefore involve family members to attain common goals for rehabilitation, such as participation in ADLs [18].

In our previous research [19], our findings stressed that it was important that people with stroke had “significant experiences that contributed to change” by performing activities they wanted and appreciated during their rehabilitation process. Therefore, activities that are relevant and valued for people in everyday life can be used as goals to improve ADL functioning [20]. Furthermore, if the people succeed in performing activities of their choice, their confidence in their capability to perform activities will be strengthened (ie, increased sense of self-efficacy) [21,22]. In our previous qualitative studies, the importance of including the client’s perspective as the point of departure for interventions has been expressed by persons with stroke as well as by HCPs [23,24]. Moreover, family support and engaging activities were described as increasing participation and functioning in everyday life [19]. The rehabilitation program F@ce 2.0 is therefore family-centered, that is, it includes both the person with stroke and his or her family members, which is in line with client-centered practice [25,26].

Mobile phones are part of information and communication technology (ICT), that is, according to the United Nations Educational, Scientific and Cultural Organization, they are “forms of technology that are used to transmit, process, store, create, display, share or exchange information by electronic means” [27]. Mobile phones have rapidly become an integral part of everyday living for people in sub-Saharan Africa and are often the sole means of communication with those living outside urban areas. According to The Mobile Economy Sub-Saharan Africa 2020 report, mobile services are predicted to continue growing in the region at an annual rate of 4.3% until 2025 [28]. Mobile phones have the potential to extend the reach of health care and rehabilitation [29] with an impact on the United Nations’ Sustainable Development Goal 3 [30], which seeks to ensure healthy lives and promote well-being for all at all ages. It has been shown that despite having physical and cognitive impairments, people with stroke could benefit from using mobile phone technology in their daily lives [31]. In addition, our previous studies [32,33] in Uganda have shown that mobile phone technology can be used to support the rehabilitation process after stroke via follow-up calls, SMS text message reminders, and feedback on the performance of activities. However, in our feasibility study of the mobile phone–supported rehabilitation program F@ce 1.0 [33], participants were recruited from an urban area, and the sample size was not powered to draw conclusions about the effects. Furthermore, F@ce 1.0 has been developed into an interdisciplinary intervention, F@ce 2.0 [34]. Hence, F@ce 2.0, which includes (1) educational workshops for the assigned HCPs, (2) a goal-directed and mobile phone–supported intervention, and (3) a web-based platform providing daily SMS text message reminders to patients, will be evaluated in a larger trial.

### Objectives

The overall aim of this project is to implement F@ce 2.0, a mobile phone–supported and family-centered rehabilitation program, and evaluate its effects on performance in daily activities and participation in everyday life in comparison to ordinary rehabilitation among persons with stroke and their family members. An additional aim is to explore the experiences of participating in F@ce 2.0 and plausible mechanisms of impact that might explain the potential effects of using F@ce 2.0 by studying the implementation process in both urban (Kampala, the capital city) and rural (Greater Masaka) areas.

Our hypothesis is that persons with stroke who participate in the F@ce 2.0 intervention will increase their functioning in daily activities and participation in everyday life as well as perceive their performance and satisfaction in daily activities and their self-efficacy to be higher than those receiving ordinary rehabilitation.

### Research Questions

We developed the following research questions (RQs):

- RQ 1: Are there any differences in effects after the 8-week intervention and at follow-up at 6 months between people with stroke who participated in F@ce 2.0 and those who received ordinary rehabilitation with regard to perceived performance in daily activities, self-efficacy, participation in everyday life, and independence in ADLs?
- RQ 2: Are there any differences after the 8-week intervention and at follow-up at 6 months between the family members of those receiving the F@ce 2.0 intervention in comparison to the family members of those receiving ordinary rehabilitation regarding caregiver burden and life satisfaction?
- RQ 3: How do the persons with stroke and their family members experience their participation in F@ce 2.0?
- RQ 4: How can the outcomes of F@ce 2.0 be understood and explained when considering the implementation process and the plausible potential mechanisms of impact?

## Methods

### Study Design

A randomized controlled trial with a pretest-posttest parallel design with an intervention group (IG) and a control group (CG) has been chosen to compare outcomes between participants receiving F@ce 2.0 (IG) and those receiving ordinary rehabilitation (CG). The study follows the Medical Research Council’s guidance [35] for evaluating complex interventions; hence, the study uses both quantitative and qualitative methodologies and a process evaluation. The trial will be monitored according to the CONSORT (Consolidated Standards of Reporting Trials) 2010 statement for nonpharmacological trials [36] and the CONSORT 2010 extension for pragmatic trials in health care [37]. Furthermore, this protocol adheres to the SPIRIT (Standard Protocol Items: Recommendations for Interventional Trials) 2013 statement [38,39]. The study has been registered at ClincialTrials.gov (NCT04337034).

### Study Setting

The F@ce 2.0 program will be implemented in 2 designated geographic areas in Uganda: Kampala (representing an urban area) and Greater Masaka (a rural area). Rehabilitation professionals or HCPs working at health care centers and CBR centers in these 2 geographic areas will be recruited to provide the F@ce 2.0 intervention or the ordinary rehabilitation intervention. These centers are funded either publicly or privately [40]. Staff numbers vary at these centers, and the recruitment of HCPs will differ depending on which types of professionals are employed. Preference will be given to occupational therapists, physiotherapists, and nurses, but other professionals might also be approached. HCPs assigned to deliver the F@ce 2.0 intervention will participate in educational workshops provided by the research group to prepare for delivering the intervention to the IG participants.

### Eligibility Criteria

Persons with stroke, family members of the persons with stroke, and HCPs working at the participating centers will be eligible to participate.

Persons with stroke will be included if the following criteria are fulfilled: (1) stroke diagnosis confirmed by computer tomography scan or by clinical symptoms; (2) enrolled at 1 of the 3 participating centers; (3) aged >18 years; (4) having no psychiatric diagnosis; (5) able to understand and formulate activity goals in English or Luganda; (6) access to, and self-reported ability to use, a mobile phone; and (7) a modified Rankin Scale level ranging from 2 to 4, indicating a slight to moderately severe disability [41].

A family member, identified as close to the person with stroke (ie, wife, husband, friend, daughter, neighbor, etc) and chosen by the person with stroke, will also be invited to participate.

### The F@ce 2.0 Program in the Ugandan Context

#### Workshops for Intervention Providers

The HCPs (ie, occupational therapists, physiotherapists, and nurses; n=4) assigned to deliver the intervention in the Kampala and Greater Masaka areas will participate in preparatory training workshops. The workshops will be organized on 2 days in 1 week as face-to-face seminars led by the research team. During the workshop sessions, the empirical and theoretical underpinnings of F@ce 2.0 will be presented and discussed. Further relevant issues regarding rehabilitation after stroke will be reflected on and discussed between the HCPs and the researchers. Furthermore, the use of ICT as a tool within rehabilitation will be initiated, discussed, and practiced during the workshop [33]. Finally, ways to integrate the F@ce 2.0 intervention into ordinary rehabilitation will be considered with the HCPs, and client cases will be used and elaborated on to facilitate their reflective learning process.

#### The F@ce 2.0 Intervention

The 8-week mobile phone–supported and family-centered intervention aims to increase functioning in daily activities for persons living with the consequences of stroke and participation in everyday life for persons with stroke and their family members. Using the Canadian Occupational Performance Measure (COPM) [42], the person with stroke formulates 3 *targets* (goals) in daily activities that they want and need to perform in the home environment. The participant, together with an identified family member, will be introduced to a problem-solving strategy framed as *target-plan-perform-prove*, intended to facilitate the learning and problem-solving process. Each activity will be practiced together with the family member, and the performance will be discussed together with the HCP to identify and formulate a *plan* for overcoming the difficulties in performing the activities chosen as targets. Different strategies will be developed and formulated together with the HCP, such as finding new ways to perform the target activities and to modify the environmental demands. To enable the family member to support the person with stroke to *perform* and practice if needed, they will be informed about the participant’s target activities and the planned strategies.

#### The Web Platform and Use of the Mobile Phone in F@ce 2.0

The participants will practice the target activities in their home environment, supported via mobile phone calls and SMS text messages. The SMS text messages will be sent from a web platform (developed using Node.js/PostgreSQL for the backend and HTML/CSS/JavaScript for the front-end) [43] where the HCPs will register the participants’ target activities and strategies for the training that they have agreed on. The participants or the family members will receive individual SMS text messages containing the 3 targets twice daily, morning and evening. The morning message will remind the participants to perform the activities during the day. In the evening, the participants, with or without support from a family member, will be asked to rate (on a scale ranging from 0 to 5) their performance of the 3 target activities, where 0=*has not performed the activity* and 5=*carried out the activity well*. If participants rate their performance as 0 or do not reply to the SMS text message reminder, a red flag alert will automatically be generated on the HCP’s mobile phone. The HCP will contact the participant the following morning to find out what happened and come to an agreement regarding the next strategy. The participants receiving F@ce 2.0 will also receive mobile phone calls as a follow-up strategy from their HCP twice a week.

#### Additional Intervention for Study Participants

All participants in the study (IG+CG) will be given oral and written information about stroke during the initial assessments. Self-reported use of health services will be collected, with assistance from family members if needed. The participants in the CG will receive the health care intervention and rehabilitation provided by their center, but they will not receive any rehabilitation supported via SMS text messages.

### Outcome Data

The primary outcomes for persons with stroke are perceived performance in daily activities evaluated with the COPM [42] and self-efficacy in performing daily activities (ie, the persons’ confidence in their ability) measured using the Self-Efficacy Scale (SES) [44,45]. The secondary outcomes will be the perceived impact of stroke evaluated with the Stroke Impact Scale 3.0 (SIS 3.0; Ugandan version) [46] and independence and dependence in ADLs measured using the Barthel Index (BI) [47]. The outcomes for family members will be caregiver burden assessed with the Caregiver Burden Scale (CBS) [48] and life satisfaction assessed with the Life Satisfaction Questionnaire-11 (LiSat-11) [49].

### Participant Timeline

Participant enrollment was initiated in 2022, and the last qualitative interview was performed during spring 2023. During this period, 90 persons with stroke and their family members were enrolled in the study. HCPs from the rehabilitation teams were enrolled as participants during the F@ce 2.0 intervention and will be followed until the study is completed.

For each participant, demographic data and baseline assessments were conducted during the first week after enrollment and postintervention assessments approximately 9 weeks after enrollment (ie, within 1 week after finalizing the program).

The study timeline is presented in Table 1.

**Table 1:** Participant timeline and data collection.

		Study period					
		Enrollment	Allocation	After allocation			
		Wk 0	Baseline	Intervention (wk 1-8)	Wk 9	6 mo	After last participant completes intervention
**Enrollment**							
	Eligibility screening	✓					
	Informed consent	✓					
**Interventions**							
	F@ce 2.0			✓			
	Control: rehabilitation as usual			✓			
**Assessments: persons with stroke**							
	Demographics		✓				
	SSS^a^		✓				
	Barthel Index		✓				
	Self-Efficacy Scale		✓		✓	✓	
	COPM^b^		✓		✓	✓	
	SIS 3.0^c^ (Ugandan version)		✓		✓	✓	
	Use of health care and risk factors		✓			✓	
	Qualitative interviews				✓	✓	
	Survey regarding intervention (Multimedia Appendix 1)				✓		
**Assessments: family members**							
	Demographics		✓				
	LiSat-11^d^		✓		✓	✓	
	Caregiver Burden Scale		✓		✓	✓	
	Qualitative interviews				✓	✓	
	Survey regarding intervention (Multimedia Appendix 1)				✓		
**Assessments: team members and HCPs^e^**							
	Demographics		✓				
	Team members’ and HCPs’ reflections after workshop and coaching	✓	✓				
	Team members’ and HCPs’ logbooks on follow-up calls and additional services supplied			✓			
	Qualitative interviews of HCPs on intervention process (Multimedia Appendix 2)						✓
	Registrations on the web platform			✓			
	Fidelity check^f^			✓	✓		

^a^SSS: Scandinavian Stroke Scale.

^b^COPM: Canadian Occupational Performance Measure.

^c^SIS 3.0: Stroke Impact Scale 3.0.

^d^LiSat-11: Life Satisfaction Questionnaire-11.

^e^HCP: health care professional.

^f^Conducted monthly with the whole team throughout the study.

### Sample Size and Power Considerations

A power calculation based on the results from our pilot study in Uganda [33] regarding the primary outcome—a clinically important difference of 2 points according to the COPM (ie, perceived performance and satisfaction in daily activities)—showed that 36 participants in each group are needed. Considering an attrition rate of 25%, a total of 90 participants will be required (α set at .05 and β at .80).

### Recruitment and Informed Consent

HCPs assigned as data collectors will identify potential study participants who meet the inclusion criteria from hospital wards, community health care units, rehabilitation centers, or physiotherapy clinics and inform them verbally about the study. The HCPs will also inform them that participation is voluntary and that they have the right to withdraw from the study at any time. In addition, the HCPs will provide participants with written information and an informed consent form in English or, when appropriate, in Luganda. Each participant will sign a consent form for voluntary participation, which emphasizes their right to withdraw from the study at any time, in accordance with the Helsinki Declaration [50]. Before patients are included, a randomization sequence will be created by the second and last authors using a random number table developed by a researcher at Karolinska Institutet in Solna, Stockholm County, Sweden. After completion of the baseline assessment, the researcher in Uganda (JK) will contact the researchers at Karolinska Institutet to obtain information on group allocation. If a participant is allocated to the IG, the researcher in Uganda (JK) will contact the HCP and inform them to provide the intervention.

The participants who consent to take part in the study will be asked to identify a family member to enroll in the study. Potential participating family members will receive verbal and written information, and written informed consent will be obtained from those who agree to participate.

### Data Collection

After informed consent is obtained, demographic data collection and baseline assessments will be performed by data collectors (HCPs at the 3 participating centers). The data collectors are not involved in delivering the intervention and are blinded to the allocation of the intervention. All data collectors will receive training before data collection to standardize data collection procedures. All information will be collected face-to-face. Demographic data (Multimedia Appendix 3) and baseline assessments will be conducted after enrollment, and follow-up assessments will be conducted within 1 week after the intervention ends and at 6 months after inclusion, preferably by the same assessors.

### Primary Outcomes

#### Persons With Stroke

The COPM assesses an individual’s perceptions of performance and satisfaction in valued daily activities within the areas of self-care, productivity, and leisure [38]. For the initial evaluation, the COPM starts with a semistructured interview lasting 30 to 40 minutes during which the persons with stroke identify activities in everyday life that they consider to be important but difficult to perform. Each activity is documented, and the participants rate the importance of each activity on a 10-point scale. They are then asked to choose 3 relevant activities and rate their performance and satisfaction with performance in each activity on separate scales, where a higher score indicates greater importance, better performance, and greater satisfaction. For the re-evaluation at the end of the intervention period, the participants are again asked to rate their performance and satisfaction in each activity. A difference of ≥2 points between the 2 evaluations indicates a clinically significant change [42]. The internal consistency of the COPM is within a reasonable range, and test-retest reliability is well above acceptable levels. Furthermore, the validity of the COPM as a measure of occupational performance has been supported by a large number of studies [51].

The SES assesses self-efficacy (ie, individuals’ confidence in their ability). The social cognitive theory formulated by Bandura [44] is the base for self-efficacy and reflects an individual’s belief in their capability to perform activities to attain a desired result. The theory of self-efficacy proposes that the stronger a person’s efficacy expectations are, the more likely the person will start and continue performing these given activities. The persons with stroke will be instructed during the interview to rate how confident they are about performing each of 16 everyday activities on a 10-point rating scale ranging from 1=*not confident at all in my ability* to 10=*very confident in my ability*. The rating process takes approximately 10 minutes, and the responses are summarized into a total score. The SES (Multimedia Appendix 4) has been adapted from a similar scale for people with pain [52] and has been used in our previous feasibility study [33].

#### Family Members

The CBS will be used to describe the perceptions and characteristics of caregiver burden among the family members assisting the person with stroke [48].

The CBS assesses 22 items, reflecting factors that include the family members’ health, general strain, isolation, disappointment, emotional involvement, and environmental aspects. The measure is administered as a self-report questionnaire and takes 20 to 30 minutes to complete. Each item is rated on a 4-point scale (1=*not at all*, 2=*seldom*, 3=*sometimes*, and 4=*often*), with a higher score indicating a greater burden. The ratings on the items are summarized into a total score. No cutoff score is used. The measure has good construct validity and test-retest reliability [48].

### Secondary Outcomes

#### Persons With Stroke

The SIS 3.0 (Ugandan version) [46] will assess the self-perceived impact of stroke in 8 domains: strength, memory and thinking, emotions, communication, ADLs and instrumental ADLs, mobility, hand function, and participation. The SIS 3.0 (Ugandan version) includes 59 items within these 8 domains, and it is administered as a structured interview that takes approximately 30 minutes to complete. Aggregated scores for each domain are generated ranging from 0 to 100; the higher the score, the lower the perceived impact of stroke (ie, fewer problems in everyday life).

Most domains of the SIS 3.0 (Ugandan version) satisfy important criteria for rating scale functioning. The internal consistency and discriminant validity are satisfactory for use among people with stroke in Uganda [46].

The Scandinavian Stroke Scale will be used to describe clinical characteristics, assess neurological impairment, and define stroke severity. This scale is administered face-to-face and takes approximately 10 minutes to complete. It provides a score for stroke severity between 2 and 5 grades of deficit ranked in decreasing order based on the level of consciousness, eye movement, orientation, speech, hand and leg movement, gait, and facial paralysis; the lower the score, the worse the deficit. The interobserver reliability for the different components is very good, as is the agreement between the domains [53].

The BI will be used to assess independence or dependence in ADLs across 10 self-care and mobility activities [47]. The values assigned to each item (0, 5, 10, or 15) are based on the time and amount of physical assistance required if the person cannot perform the activity independently. Scores range from 0 to 100, with a lower score indicating greater dependency. The BI is administered through an interview that takes approximately 10 minutes to complete and is reliable and valid for people with stroke [54].

#### Family Members

The LiSat-11 will be used to measure global life satisfaction as well as satisfaction across 10 domains using a 6-point scale [49]. The LiSat-11 is completed through self-report, which takes approximately 10 minutes. The score on each domain is dichotomized and presented without summarizing the overall scores. The questionnaire has demonstrated acceptable test-retest reliability, specificity, and sensitivity [49].

Health care use will be collected through a structured interview with the person with stroke, together with the family member.

### Experiences of Taking Part in F@ce 2.0 Among Persons With Stroke and Their Family Members

Semistructured qualitative interviews (Multimedia Appendix 2) will be conducted face-to-face with persons with stroke and their family members. Interviews with the person with stroke (n=6-10) and family members (n=6-10) from both rural and urban areas will be conducted in their home setting after they complete the F@ce 2.0 intervention. Their experiences of participating in F@ce 2.0 will be explored, and the data from the interviews will be analyzed using a grounded theory approach [55]. The participant group will be selected to represent a range of experiences (eg, varied characteristics regarding sex, level of disability, and living conditions) to ensure the collection of rich qualitative data. An interview guide with open-ended questions focusing on participants’ everyday life experiences when participating in the F@ce 2.0 intervention will be used. Field notes will be taken during the interviews, and all interviews will be digitally recorded and transcribed verbatim and translated into English if conducted in Luganda. The transcripts from these interviews will also be used in the process evaluation described in the next subsection.

### Process Evaluation of F@ce 2.0 in the Ugandan Context

A process evaluation is essential for designing and evaluating complex interventions, such as F@ce 2.0, and refers to activities related to the implementation, acceptance, and reach of an intervention. A process evaluation aims to answer questions about how the intervention interacts with its context, how different perspectives from stakeholders can be integrated, or how the intervention can be refined [56]. This study will use a single-case study design with mixed methods, combining qualitative and quantitative data to explore the implementation process and mechanisms of impact of the F@ce 2.0 program. Quantitative process data will include metrics such as the mean number of minutes per session with clients, the number of telephone contacts between HCPs and clients, and data from the web platform. The HCPs’ logbooks will also be collected. In addition, semistructured interviews (Multimedia Appendix 2) will be conducted with persons with stroke, their family members, and the HCPs providing the intervention. The interviews with the persons with stroke and family members will focus on exploring their perceptions of the value, benefits, and detrimental or unintended consequences of the intervention, as well as their perspectives of the intervention’s acceptability, fidelity, reach, and dose. The interviews with HCPs will cover their reflections and reasoning in relation to preparations for delivering the intervention at the workshops, as well as the intervention delivery process, aiming to explore the implementation of the intervention. The logbooks maintained by the HCPs will include their field notes and reflections after the workshop and coaching sessions. These field notes will describe conditions that facilitated or hindered session delivery, as well as potential positive or negative side effects. The HCPs’ acceptability of the intervention will also be documented. From the web platform, information on response rates to SMS text messages as well as target achievement will be derived.

### Data Analyses

Anonymized data will be entered into SPSS for Windows (IBM Corp) [57]. The number of participants (persons with stroke and family members) being recruited will be presented in a flowchart according to the CONSORT statement [36,37]. The retention rate and adherence to the intervention (eg, responses to SMS text messages, HCPs’ follow-up meetings with the participants, and all other services related to the intervention, as well as the number of participants seen by each HCP or team) will be presented based on frequencies and percentages. Descriptive statistics will be used to present the characteristics of the participants, health care use, and the outcomes after the intervention and at 6 months.

All statistical analyses comparing the primary and secondary outcomes between the IG and the CG will be performed using intention-to-treat analysis [58]. For missing values, the imputation method of last value carried forward will be used. To assess differences in changes in primary outcomes between the IG and CG at the different data collection points, multivariate statistical methods will be used. Covariates will include sex, age, stroke severity, and independence and dependence in ADLs before stroke, as measured by the BI. The statistician will be blinded to the group allocation for the intervention.

The interviews with persons with stroke and their family members will explore their experiences of participating in F@ce 2.0, and we will analyze the interview data using a grounded theory approach [55]. The interviews will be transcribed verbatim. The interview material will be analyzed through constant comparison of the content in the transcripts. The material will be read several times to get to know the data profoundly. Next, the initial coding will be performed by coding the material line by line, followed by focused coding where the initial codes from each interview will be compared. In the next step, the codes will be compiled into categories and subcategories. The findings will present a meaning structure comprising a core category and subcategories of the studied phenomenon, that is, the meaning of participating in a mobile phone–supported and family-centered intervention and the implications for everyday life after stroke [55]. The qualitative data used in the process evaluation will be analyzed using content analysis [59]. In this analysis, meaning-bearing units will be identified, condensed, and abstracted to codes from each interview. The codes will be compared for similarities and differences and organized into categories and subcategories that will describe an abstract level of the content of all interviews [59].

All personal information (eg, names) will be removed during transcription. Copies of the digital recordings will be destroyed once transcription is complete. Interview transcriptions and all other data will be coded and stored in a secure electronic database.

### Evaluation of Outcomes

The changes in performance and satisfaction in the 3 stated valued activities as perceived by the person with stroke will be assessed using the COPM scores. The 2 summative COPM scores—one for performance and one for satisfaction—will be divided by the number of chosen and rated activities to generate scores for comparisons across time and between groups [42].

The confidence in performance of daily activities as perceived by the person with stroke will be presented based on the SES scores [33].

All data regarding the BI and SIS 3.0 (Ugandan version) for the persons with stroke and the data for the family members will be analyzed and reported according to the norms of the measures [46,54].

### Experiences of Daily Life of the Participants When Taking Part in F@ce 2.0

A grounded theory approach will be applied [55] for analyzing the qualitative interviews and discerning core categories and subcategories constituting the findings regarding the participants’ experiences of daily life when taking part in F@ce 2.0.

### Process Evaluation: Process Data and Qualitative Interviews

Both quantitative and qualitative data will be integrated in a mixed methods analysis to explain the outcomes of the F@ce 2.0 intervention, the implementation process, and the potential mechanisms of impact. Constant comparison [59] will be used to analyze the semistructured interviews from the persons with stroke, their family members, and the HCPs describing (1) the perceived value, benefits, and detrimental or unintended consequences of the intervention (ie, perceived harm); (2) the acceptability of the intervention in practice; and (3) the fidelity, reach, and dose of the intervention. All quantitative data will be analyzed using descriptive statistics. Data sources used to capture the process of implementation, contexts, and mechanisms of impact in different ways will be analyzed separately at first and integrated in a joint analysis thereafter to explore the relationships between the findings. The analysis of the data on context will be guided by the Promoting Action on Research Implementation in Health Services framework (the context component) [60].

### Patient and Public Involvement

The feasibility study for F@ce 1.0 [33] and the experiences of family members participating in the intervention conducted in Uganda [61] provided important information for the continued modeling of the intervention. Furthermore, the process evaluation of F@ce 1.0 in the Ugandan context [62] included the experiences of health care staff, managers in health care, family members, persons with stroke, and technicians, which added to the knowledge base and informed the choice of RQs, design, and delivery of the studies that made up the research project. The modeling of F@ce 2.0 and its contextual adaptation, together with the results of this study, will be presented to, and discussed with, various stakeholders at regional and national levels as well as private rehabilitation clinics and CBR providers.

### Ethical Considerations

The study will be performed in accordance with the Declaration of Helsinki, which outlines ethical principles for medical research [50], and has been approved by the Mulago Hospital Research and Ethics Committee for the intervention in urban Kampala (00941; February 26, 2021) and by the Masaka Regional Referral Hospital Research Committee for the intervention in rural Masaka (ADM. 170106; April 13, 2021). The Uganda National Council for Science and Technology has approved the entire project (HS1528ES; January 19, 2022).

All participants will sign an informed consent form of voluntary participation, which emphasizes their right to withdraw from the study at any time. A copy of the form will be provided to the participants. Withdrawal from the study will be recorded by the researchers. Each participant (participants with stroke, significant others, and HCPs) will receive an ID number. The data will be deidentified; hence, the analysis will be performed and the results presented confidentially. Each participant will be compensated 10,000 Ugandan shillings (US $2.71) for their time at each data collection point.

## Results

As of March 2024, a total of 100 participants with stroke had been enrolled in the randomized controlled trial, of whom 2 (2%) died of natural causes. A total of 96 family members had also been enrolled. The intervention has been performed via daily reminders sent by SMS text messages and phone calls twice a week by the interventionist. Data collection was initiated on January 25, 2022. All baseline and follow-up assessments have been completed. Data analysis is planned to start in October 2024 for the randomized controlled trial and it is planned to be submitted during May 2025. Data collection for the qualitative study and the process evaluation have been completed. Data analysis for the qualitative study will be initiated in February 2025. The process evaluation has been submitted to a scientific journal.

## Discussion

### Summary

To our knowledge, this is the first project to include a full-scale evaluation of an ADL intervention in sub-Saharan Africa and where we will be able to demonstrate its impact. The feasibility of this intervention has been tested [33], and we are ready to take the next step. The purpose of this research project is therefore to implement and evaluate F@ce 2.0 with the intention of enabling functioning in ADLs and participation in everyday life for people with stroke and their families. Our hypothesis is that people with stroke who participate in the F@ce 2.0 intervention will increase functioning in daily activities and participation in everyday life as well as perceive their performance and satisfaction in daily activities and their self-efficacy to be higher than those of people who receive ordinary rehabilitation. The project’s goals are highly relevant because they align with the United Nations’ Sustainable Development Goals 2030, including goals to reduce the impact of noncommunicable diseases and to ensure healthy lives and promote well-being for all at all ages.

### Building on Our Previous Feasibility Study

The previously completed feasibility study conducted in Kampala showed that the mobile phone–supported and family-centered F@ce program could, with some technical adjustments, be useful in rehabilitation [33]. The design used could be replicated in a larger trial, but improvements in recruitment, allocation concealment, randomization, and blinding of data collectors were needed. Overall, the results indicated the need for further research, which should also include participants from rural areas. Therefore, the F@ce program, in collaboration with local researchers as well as health care and rehabilitation professionals, has now been modeled to fit the actual context and is expected to positively impact stroke rehabilitation in Uganda. It is also aligned with the overarching goal of Swedish development aid to contribute to environments that support people with low socioeconomic status in their efforts to improve their quality of life.

### Strengths and Limitations

Uganda has a population of 45 million, with 23% living in urban areas. The country is characterized by generally weak infrastructure, such as bad roads, poor transport systems, inadequate electricity supply, few health care units, and limited numbers of rehabilitation professionals. However, Uganda has witnessed a remarkable growth in mobile phone use, with a penetration rate of 67% as of February 2020, according to the Uganda Communications Commission. Approximately 20 million people in Uganda, representing 44% of the population, have a mobile phone subscription. Advances in technology have transformed mobile phones into all-in-one devices that can be used almost anywhere. Nearly half of all mobile subscribers also access mobile internet services. A strength of F@ce 2.0 might be the use of mobile phones to provide digital support for daily activities after stroke, which might help deliver rehabilitation services in a context with limited resources and staffing. A limitation that we have seen in our previous studies [17,34] could be that it is difficult to check and control for whether the participants have received other rehabilitation services or support that contributed to a change. Furthermore, we are aware that many of the instruments we use are self-rated or -reported instruments and that it is the participants themselves who will provide their responses via telephone to the data collectors. However, the instruments have been well tested and used in our earlier feasibility studies [17,34]. This approach was selected to make it possible to also include people living in rural Uganda.

It is expected that this research, using a randomized controlled trial design and including people from both urban and rural areas, will contribute with knowledge of the effects of the F@ce 2.0 intervention program. Furthermore, the process evaluation will provide knowledge important for future practice, particularly regarding the value, acceptability, and fidelity of the intervention.

### Dissemination

Dissemination efforts will include scientific publications in open-access, peer-reviewed journals and presentations at national and international conferences, as well as reports to funders. The results will also be presented to staff and decision makers at the municipalities involved in the study, to the Ugandan public through press releases and articles in the daily press, and at conferences and fairs focused on technical solutions. A comprehensive program with suggestions for ways to implement F@ce 2.0 will be prepared for organizations such as those representing rehabilitation services (nongovernmental organizations and patient and stakeholder organizations).

### Conclusions

This research study presents a unique and highly relevant opportunity to enhance knowledge and address gaps in stroke rehabilitation. The study will yield robust data on the intervention’s effectiveness as well as in-depth insights into the contextual factors and mechanisms influencing its impact. Health care providers participating in the study will acquire resources to engage patients and families with digital support through mobile phones, fostering interprofessional skills in using ICT within rehabilitation. This will enable them to reach their patients in both rural and urban areas, meet patient needs, and improve rehabilitation support in the Ugandan context.

## Appendix

Multimedia Appendix 1 Questions on the intervention for persons with stroke and family members.

Multimedia Appendix 2 Interview guides for the process evaluation and qualitative study.

Multimedia Appendix 3 Demographics of persons with stroke and family members.

Multimedia Appendix 4 Self-Efficacy Scale.

Multimedia Appendix 5 Peer-review report by Vetenskapsrådet - The Swedish Research Council (Sweden).

## Abbreviations

ADL: activity of daily living
BI: Barthel Index
CBR: community-based rehabilitation
CBS: Caregiver Burden Scale
CG: control group
CONSORT: Consolidated Standards of Reporting Trials
COPM: Canadian Occupational Performance Measure
HCP: health care professional
ICT: information and communication technology
IG: intervention group
LiSat-11: Life Satisfaction Questionnaire-11
RQ: research question
SES: Self-Efficacy Scale
SIS 3.0: Stroke Impact Scale 3.0
SPIRIT: Standard Protocol Items: Recommendations for Interventional Trials

## Data Availability

Data sharing is not applicable to this paper as no data sets were generated or analyzed during this study.

## References

[1] (2021). WHO policy on disability. World Health Organization (WHO).

[2] Townsend E, Polatajko JH (2007). Enabling Occupation II: Advancing an Occupational Therapy Vision for Health, Well-being, and Justice Through Occupation.

[3] Eide AH, Josephsson S, Vik K (2017). Participation in Health and Welfare Services: Professional Concepts and Lived Experience.

[4] (2018). Nationella riktlinjer – Utvärdering: Vård vid stroke: Huvudrapport med förbättringsområden. Socialstyrelsen.

[5] Stroke, cerebrovascular accident. World Health Organization.

[6] Lindsay MP, Norrving B, Sacco RL, Brainin M, Hacke W, Martins S, Pandian J, Pandian V Global stroke fact sheet 2019. World Stroke Organization (WSO).

[7] Owolabi MO, Akarolo-Anthony S, Akinyemi R, Arnett D, Gebregziabher M, Jenkins C, Tiwari H, Arulogun O, Akpalu A, Sarfo FS, Obiako R, Owolabi L, Sagoe K, Melikam S, Adeoye AM, Lackland D, Ovbiagele B (2015). The burden of stroke in Africa: a glance at the present and a glimpse into the future. Cardiovasc J Afr.

[8] Urimubenshi G, Cadilhac DA, Kagwiza JN, Wu O, Langhorne P (2018). Stroke care in Africa: a systematic review of the literature. Int J Stroke.

[9] Akinyemi RO, Ovbiagele B, Adeniji OA, Sarfo FS, Abd-Allah F, Adoukonou T, Ogah OS, Naidoo P, Damasceno A, Walker RW, Ogunniyi A, Kalaria RN, Owolabi MO (2021). Stroke in Africa: profile, progress, prospects and priorities. Nat Rev Neurol.

[10] Global health - Uganda. Centers for Disease Control and Prevention.

[11] Legg L, Drummond A, Leonardi-Bee J, Gladman JR, Corr S, Donkervoort M, Edmans J, Gilbertson L, Jongbloed L, Logan P, Sackley C, Walker M, Langhorne P (2007). Occupational therapy for patients with problems in personal activities of daily living after stroke: systematic review of randomised trials. BMJ.

[12] Where we work: Uganda. Opportunity International.

[13] (2018). Data: rural population (% of total population). The World Bank.

[14] Khasnabis C, Heinicke Motsch K (2010). Community-based rehabilitation: CBR guidelines. World Health Organization (WHO).

[15] Oni OD, Olagunju AT, Okpataku CI, Erinfolami AR, Adeyemi JD (2019). Predictors of caregiver burden after stroke in Nigeria: effect on psychosocial well-being. Indian J Psychiatry.

[16] Zhu W, Jiang Y (2018). A meta-analytic study of predictors for informal caregiver burden in patients with stroke. J Stroke Cerebrovasc Dis.

[17] Kamwesiga JT (2018). The Perceived Impact of Stroke and Feasibility of a Mobile Phone Supported ADL Intervention in Uganda [thesis].

[18] Levack WM, Siegert RJ, Dean SG, McPherson KM (2009). Goal planning for adults with acquired brain injury: how clinicians talk about involving family. Brain Inj.

[19] Guidetti S, Eriksson G, von Koch L, Johansson U, Tham K (2022). Activities in daily living: the development of a new client-centred ADL intervention for persons with stroke. Scand J Occup Ther.

[20] Kristensen HK, Persson D, Nygren C, Boll M, Matzen P (2011). Evaluation of evidence within occupational therapy in stroke rehabilitation. Scand J Occup Ther.

[21] Bandura A (1977). Self-efficacy: toward a unifying theory of behavioral change. Psychol Rev.

[22] Bandura A, Adams NE, Beyer J (1977). Cognitive processes mediating behavioral change. J Pers Soc Psychol.

[23] Gustavsson M, Ytterberg C, Nabsen Marwaa M, Tham K, Guidetti S (2018). Experiences of using information and communication technology within the first year after stroke - a grounded theory study. Disabil Rehabil.

[24] Marwaa MN, Ytterberg C, Guidetti S (2020). Significant others' perspectives on person-centred information and communication technology in stroke rehabilitation - a grounded theory study. Disabil Rehabil.

[25] Bertilsson AS, Ranner M, von Koch L, Eriksson G, Johansson U, Ytterberg C, Guidetti S, Tham K (2014). A client-centred ADL intervention: three-month follow-up of a randomized controlled trial. Scand J Occup Ther.

[26] Guidetti S, Ranner M, Tham K, Andersson M, Ytterberg C, von Koch L (2015). A "client-centred activities of daily living" intervention for persons with stroke: one-year follow-up of a randomized controlled trial. J Rehabil Med.

[27] The UNESCO ICT in education programme. United Nations Educational, Scientific and Cultural Organization (UNESCO).

[28] GSMA publishes 2020 the mobile economy sub-saharan Africa report. The Digital Watch Observatory.

[29] Hellström J (2010). The innovative use of mobile applications in East Africa. Mobile World Live.

[30] The 17 goals. Department of Economic and Social Affairs, United Nations.

[31] Larsson Lund M, Lövgren-Engström AL, Lexell J (2011). Using everyday technology to compensate for difficulties in task performance in daily life: experiences in persons with acquired brain injury and their significant others. Disabil Rehabil Assist Technol.

[32] Kamwesiga JT, Tham K, Guidetti S (2017). Experiences of using mobile phones in everyday life among persons with stroke and their families in Uganda - a qualitative study. Disabil Rehabil.

[33] Kamwesiga JT, Eriksson GM, Tham K, Fors U, Ndiwalana A, von Koch L, Guidetti S (2018). A feasibility study of a mobile phone supported family-centred ADL intervention, F@ce™, after stroke in Uganda. Global Health.

[34] Guidetti S, Gustavsson M, Tham K, Andersson M, Fors U, Ytterberg C (2020). F@ce: a team-based, person-centred intervention for rehabilitation after stroke supported by information and communication technology - a feasibility study. BMC Neurol.

[35] Craig P, Dieppe P, Macintyre S, Michie S, Nazareth I, Petticrew M (2013). Developing and evaluating complex interventions: the new medical research council guidance. Int J Nurs Stud.

[36] Boutron I, Altman DG, Moher D, Schulz KF, Ravaud P, CONSORT NPT Group (2017). CONSORT statement for randomized trials of nonpharmacologic treatments: a 2017 update and a CONSORT extension for nonpharmacologic trial abstracts. Ann Intern Med.

[37] Zwarenstein M, Treweek S, Gagnier JJ, Altman DG, Tunis S, Haynes B, Oxman AD, Moher D (2008). Improving the reporting of pragmatic trials: an extension of the CONSORT statement. BMJ.

[38] Chan AW, Tetzlaff JM, Gøtzsche PC, Altman DG, Mann H, Berlin JA, Dickersin K, Hróbjartsson A, Schulz KF, Parulekar WR, Krleza-Jeric K, Laupacis A, Moher D (2013). SPIRIT 2013 explanation and elaboration: guidance for protocols of clinical trials. BMJ.

[39] Chan AW, Tetzlaff JM, Altman DG, Laupacis A, Gøtzsche PC, Krleža-Jerić K, Hróbjartsson A, Mann H, Dickersin K, Berlin JA, Doré CJ, Parulekar WR, Summerskill WS, Groves T, Schulz KF, Sox HC, Rockhold FW, Rennie D, Moher D (2013). SPIRIT 2013 statement: defining standard protocol items for clinical trials. Ann Intern Med.

[40] The second national health policy: promoting people’s health to enhance socio-economic development. Ministry of Health, Republic of Uganda.

[41] Bonita R, Beaglehole R (1988). Recovery of motor function after stroke. Stroke.

[42] Law M, Carswell A, McColl MA, Pollock N, Carswell A, Polatajko H (2005). The COPM is an individualized, client-centred outcome measure. Canadian Occupational Performance Measure. 4th edition.

[43] Fors U, Kamwesiga JT, Eriksson GM, von Koch L, Guidetti S (2019). User evaluation of a novel SMS-based reminder system for supporting post-stroke rehabilitation. BMC Med Inform Decis Mak.

[44] Bandura A, Bandura A (1997). The nature and structure of self-efficacy. Self Efficacy: The Exercise of Control.

[45] Gustavsson C, Denison E, von Koch L (2011). Self-management of persistent neck pain: two-year follow-up of a randomized controlled trial of a multicomponent group intervention in primary health care. Spine (Phila Pa 1976).

[46] Kamwesiga JT, von Koch L, Kottorp A, Guidetti S (2016). Cultural adaptation and validation of stroke impact scale 3.0 version in Uganda: a small-scale study. SAGE Open Med.

[47] Mahoney F, Barthel D (1965). Functional evaluation: the Barthel index. Md State Med J.

[48] Elmståhl S, Malmberg B, Annerstedt L (1996). Caregiver's burden of patients 3 years after stroke assessed by a novel caregiver burden scale. Arch Phys Med Rehabil.

[49] Fugl-Meyer AR, Melin R, Fugl-Meyer KS (2002). Life satisfaction in 18- to 64-year-old Swedes: in relation to gender, age, partner and immigrant status. J Rehabil Med.

[50] WMA declaration of Helsinki – ethical principles for medical research involving human subjects. World Medical Association.

[51] Psychometric properties of the COPM. COPM Canadian Occupational Performance Measure.

[52] Denison E, Åsenlöf P, Lindberg P (2004). Self-efficacy, fear avoidance, and pain intensity as predictors of disability in subacute and chronic musculoskeletal pain patients in primary health care. Pain.

[53] Barber M, Fail M, Shields M, Stott DJ, Langhorne P (2004). Validity and reliability of estimating the scandinavian stroke scale score from medical records. Cerebrovasc Dis.

[54] Quinn TJ, Langhorne P, Stott DJ (2011). Barthel index for stroke trials: development, properties, and application. Stroke.

[55] Charmaz K (2014). Constructing Grounded Theory. 2nd edition.

[56] Skivington K, Matthews L, Simpson SA, Craig P, Baird J, Blazeby JM, Boyd KA, Craig N, French DP, McIntosh E, Petticrew M, Rycroft-Malone J, White M, Moore L (2021). A new framework for developing and evaluating complex interventions: update of Medical Research Council guidance. BMJ.

[57] IBM SPSS statistics for Windows. IBM Corp.

[58] Ranganathan P, Pramesh CS, Aggarwal R (2016). Common pitfalls in statistical analysis: intention-to-treat versus per-protocol analysis. Perspect Clin Res.

[59] Graneheim UH, Lundman B (2004). Qualitative content analysis in nursing research: concepts, procedures and measures to achieve trustworthiness. Nurse Educ Today.

[60] Rycroft-Malone J, Rycroft-Malone J, Bucknall T (2010). Promoting action on research implementation in health services (PARIHS). Models and Frameworks for Implementing Evidence-Based Practice: Linking Evidence to Action.

[61] Eriksson GM, Kamwesiga JT, Guidetti S (2021). The everyday life situation of caregivers to family members who have had a stroke and received the rehabilitation intervention F@ce in Uganda. Arch Public Health.

[62] Teriö M, Eriksson G, Kamwesiga JT, Guidetti S (2019). What's in it for me? A process evaluation of the implementation of a mobile phone-supported intervention after stroke in Uganda. BMC Public Health.

