# Supplementary material for: Implementing and Evaluating a Mobile Phone–Supported and Family-Centered Rehabilitation Program for People With Stroke in Uganda (F@ce 2.0): Protocol for a Randomized Controlled Trial
Source: JMIR Res Protoc. 2024 Sep 25;13:e60955. doi: 10.2196/60955 (PMC11464936; doi:10.2196/60955)
Supplement: Multimedia Appendix 1 [file resprot_v13i1e60955_app1.docx]

Questions to all persons with stroke (IG) at completed intervention (8 weeks)
CODE________________ (Persons with stroke)

| Questions regarding the training/rehabilitation related to the stroke |
| --- |
| 1. To which extent have you been taking part of the goal setting in your rehabilitation together with the interventionist?   5 to a large extent  4  3  2  1 not at all  Do not know  Not applicable – do not have any goals/target for my rehabilitation |
| 1. To which extent concerns your goals/targets in the intervention activities that are valued/important for you in your everyday life?   5 To a large extent  4  3  2  1 Not at all  Do not know  Not applicable – do not have any goals/targets for my rehabilitation |
| 1. Can you mention/describe some of the goals/targets for the training/rehabilitation?   Target 1: _______________________________________________________________  Target 2: ______________________________________________________________  Target 3: _______________________________________________________________ |
| 1. To which extent do you know how you can train on your own to recover from your stroke?   5 To a large extent  4  3  2  1 Not at all  Do not know  Not relevant to train on my own |
| 1. To which extent do you get support from your family members in your training/rehabilitation?   5 To a large extent  4  3  2  1 Not at all  Do not know  Not current with support from family members |
| 1. During a usual week, how many days do you perform your training/rehabilitation on your own?   everyday  most days  occasionally  never |
| 1. If you perform your training on your own, approximately how much time do you use for training a usual day?   0–30 minutes  30–60 minutes  1–2 hours  more than 2 hours |

1. How did you feel about using the SMSs for reminders of your targets for the rehabilitation?
   - - Positive/Good
     - Fun
     - Interesting
     - Boring
     - Negative/Bad
2. Do you think the SMS-reminders have helped you to recover from your stroke or improve in the performance of your daily activities in any way?

- Very much
  - - Much
    - A little
    - Not at all
    - Comments?.................................................................................................................................................................................................................................................................

1. Now when the intervention is over, will you miss the daily SMSs and targets?
   - - Yes
     - No

Questions to all family members) (IG) at completed intervention (8 weeks)

CODE________________(Family members)

| The following questions concern your family member´s training/rehabilitation related to his/her stroke. |
| --- |
| 1. To which extent do you provide support to your family member in his/her training after the stroke?   5 To a large extent  4  3  2  1 Not at all  Do not know  Not relevant |
| 1. Do you know the goals/targets your family member has for his/her training?   yes  partly  no  If yes – describe the targets below  Target 1: _______________________________________________________________  Target 2: _______________________________________________________________  Target 3: _______________________________________________________________ |

| 1. To which extent do you experience that your family member receives the support in the rehabilitation/training that he/she needs?   5 To a large extent  4  3  2  1 Not at all  Do not know  Not relevant with training/rehabilitation |
| --- |
| 1. To which extent do you take responsibility for your family member´s training/rehabilitation?   5 To a large extent  4  3  2  1 Not at all  Do not know  Not relevant with training/rehabilitation |

1. Do you think the SMS-reminders have helped your family member to recover from his/her stroke or improve in the performance of his/her daily activities in any way?

- Very much
  - - - Much
      - A little
      - Not at all
      - Comments?................................................................................................................................................

1. What will happen to your family member now, when the SMS-reminders has stopped to come?
   - - - He/she will lack the SMS reminders
       - There will be no change for him/her
       - He/she will be happy to not receiving the SMSs any longer
