# Supplementary material for: Implementing and Evaluating a Mobile Phone–Supported and Family-Centered Rehabilitation Program for People With Stroke in Uganda (F@ce 2.0): Protocol for a Randomized Controlled Trial
Source: JMIR Res Protoc. 2024 Sep 25;13:e60955. doi: 10.2196/60955 (PMC11464936; doi:10.2196/60955)
Supplement: Multimedia Appendix 2 [file resprot_v13i1e60955_app2.docx]

**Interview-guide for the process evaluation**

**Start-up Questions & Information**

Do you accept that I record this interview?

I would like to emphasize that you are anonymous throughout this entire study and that you can at any time decline to answer specific questions or withdraw from this study completely.

**Questions for all study participants**

- How would you describe your role and areas of responsibility in F@ce 2.0?
- Could you tell me about your experience of being a part of F@ce 2.0 intervention for stroke rehabilitation?
- In the F@ce 2.0 initiative - what has worked well and what has worked less well?
- Could you describe the implementation of F@ce 2.0, was it performed according to the manual?
- How do you think the context have affected the intervention that was implemented?
- Describe in what way your knowledge and ability matched the role you should have and when it did not match?
- Have you encountered any unintended consequences?
  - If so, do you feel you have received adequate support when encountering unintended consequences?
  - How was the support you got framed/designed?
- What problems have you experienced with F@ce 2.0?
  - What lessons can be learned from the innovation?
  - What adjustments should be made for the future?
- How do you see the resources in the project? Do you miss something?
- How would you describe the general communication and information sharing between the team members? What communication methods did you use?
- How has it been working with the technological part in this project?
- Is there anything else you would like to add that you believe we have not covered?

**Specific questions for different work role**

**Researcher**

- How has it been to project F@ce 2.0?

**Occupational therapist**

- Tell me about the workshop that all health professionals attended before the intervention started. Do you have any reflections of the workshop? If you should have given the workshop, how would you have planned it?
- Tell me about your experiences of how F@ce functioned as a tool in working with patients (how it was received and used by patients, whether and how you used the assessments/goals/COMP, whether it was supportive/motivating/positive or inconvenient/obstructive/made no difference - how, why?)
- Tell me about your experiences with F@ce and relatives (what the role of relatives looked like, how F@ce was received by relatives, how the relatives' participation/support for their loved one with stroke looked like, how (if) it was supportive/motivating/ positive - how/why?, troublesome/obstructive/made no difference - how, why?)

**Data collectors**

- How has it been working with the data collecting? How did you do that?
- How was it to do data collecting with caregivers?
- How was it to set the goals together with the patient?
- Tell me about how you reasoned when you asked patients to try F@ce 2.0 and be part of the study (which patients did you ask, which you didn't ask, if you could have asked more, which patients are suitable for F@ce?)

**Interview guide for the qualitative study** ID NO:______

**Inclusion criteria:** 1) Participated in F@ce 2.0; persons with stroke and family members

2) variation: female and males

**Aim:**

**To explore and describe the experiences of people living with stroke and family members of participating in the F@ce intervention**

**Person with stroke**

-Tell me about yourself?

***Probe for****; Age, marital status, what do you do for income, religion?*

- Tell me about your health?

***Probe for*** *stroke, When it happened*, *What did you do to address /solve this problem? When it occurred, how did you feel when it occurred, how did you come to know that it was stroke, what did you do to address the problem,*

- Tell me about your how your day-to-day activities was like before the stroke?

-What help/support did you need and which help did you get after your stroke?

***Probe for*** *who helped, i.e., family/relative/neighbour, kind of help offered?*

*Changes before/ after stroke?*

Tell me about your day-to-day activities today? Which are your greatest challenges in your daily living and how do you handle them?

-Tell me about what is important to you in your life today?

-Tell me if you miss doing something that you did before you got a stroke?

-What were your experiences in accessing this rehabilitation that you have had?

-Can you tell me about how a day looks like for you when taking part in the intervention, from when you get up in the morning until you go to bed in the evening

-Can you tell me about the first contact with anyone providing the rehabilitation?

What happened during the rehabilitation later on?

-Can you tell me about the goals/targets you have/had for your rehabilitation. How did you decide to choose these targets?

-Can you tell me about how you chose these targets to train during the rehab

-Can you tell me about what worked for you in the rehabilitation? What you liked/disliked?

-How was the contact with the OT when he called? What did you talk about? How did you talk about the training to reach the targets?

-Tell me about how it was for you to get SMS-reminders in the morning.

-How was it for you to rate how well you did the activities that was your set targets? How did you do the ratings, on your own, together with a family member, together with the OT?

-How important were the SMS reminders for you? How often did they come?

-How do you reason for the future when you get new challenges in everyday life? Have you discussed any thinking/strategies to use to solve future problems?
