# Supplementary material for: Implementing and Evaluating a Mobile Phone–Supported and Family-Centered Rehabilitation Program for People With Stroke in Uganda (F@ce 2.0): Protocol for a Randomized Controlled Trial
Source: JMIR Res Protoc. 2024 Sep 25;13:e60955. doi: 10.2196/60955 (PMC11464936; doi:10.2196/60955)
Supplement: Multimedia Appendix 3 [file resprot_v13i1e60955_app3.docx]

# Stroke rehabilitation F@CE

1. **Client-information- DEMOGRAPHIC**

1. Age:………………years
2. Gender

Male  (1)

Female  (2)

1. Which side of your body is affected?

Right  (1)

Left  (2)

1. Which hand is your dominant hand?

(right-handed)  (1)

(left-handed)  (2)

1. Highest level of education

No education  (1)

Primary school  (2)

Secondary school  (3)

Tertiary school  (4)

University  (5)

1. What is your religion?

Catholic  (1)

Anglican  (2)

Muslim  (3)

Other  (4)

If other, please specify:…………………………………………………

**The following questions refer to the everyday life now of persons’ that had had a stroke**

1. a) Do you have a source of livelihood **now?** Yes  (1) No  (2)

Business  (1)

Salary  (2)

Sale of agricultural produce  (3)

Casual labour  (4)

Others  (5) Specify others............................................................

8a) Did you have a source of livelihood **before stroke?** Yes  (1) No  (2)

8b) Business  (1)

Salary  (2)

Sale of agricultural produce  (3)

Casual labour  (4)

Others  (5) Specify others............................................................

9.a) How many people in your household have a source of livelihood (including the respondent)? …………..

1. Housing **at time of interview**

Rented house  (1)

Owned house  (2)

Rented apartment  (3)

Owned apartment  (4)

Staff quarter  (5)

Others  (6)

Others, specify……………………………………………….

1. a) Are you currently living in your own home? Yes  (1) No  (2)

b) *If* ***no****, who are you staying with?..........................................................................*

c) *If* ***no****, why have you moved?.................................................................................*

1. Housing **before stroke?**

Rented house  (1)

Owned house  (2)

Rented apartment  (3)

Owned apartment  (4)

Staff quarter  (5)

Others (6)

Others, specify…………………………………………………………………..

1. Marital status **now**?

Married, living together  (1)

Married, not living together  (2)

Single  (3)

Widow/Widower  (4)

Divorced/separated  (5)

Marital status **before stroke?**

Married, living together  (1)

Married, not living together  (2)

Single  (3)

Widow/Widower  (4)

Divorced/separated  (5)

1. How many members do you have in your household (including the respondent)?...............
2. a) How many biological children do you have?...........................................................

b) How many in the household are totally economically dependent? ….......................

c) How many in the household are partly economically dependent? ­­­................................

d) How many children (both biological and extended) below the age of 18 are living at

home?.........................

e) Do you have access to a mobile phone? Yes  (1) No  (2)

1. Were you mentally or physically incapacitated in any way **before the stroke?**

Yes  (1) No  (2)

1. Did you get any help in your daily activities from someone before the stroke?

Yes  (1) No  (2)

*If* ***yes****, from whom?* Family member (1)

Neighbour  (2)

Friend  (3)

Other  (4)

1. How old were you at stroke onset? ………………years
2. a) Do you get any help in your daily activities from someone **now?**

Yes  No

*If* ***yes****, from whom?* Family member  (1)

Neighbour  (2)

Friend  (3)

Other  (4)

b) *If* ***yes****, how often do you get help*? Daily  (1) Weekly  (2)

c) Which activities do you find difficult?..........................................................……….

...........................................................................................................................................

…………………………………………………………………………………………

1. Did you use assistive aids **before stroke?** Yes  (1) No  (2)
2. *If* ***yes****, which assistive aids did you use?*

Crutch  (1)

Cane/stick  (2)

Walker  (3)

Wheelchair  (4)

Other (5)

Other, specify………………………………………………

1. Do you use assistive aids **now?** Yes  (1) No  (2)

1. If yes, which assistive aids do you use? (tell the respondent you want to see it)

Crutch  (1)

Cane/stick  (2)

Walker  (3)

Wheelchair  (4)

Other (5)

Other, specify…………………………………………

1. Have you received any medical health care (nurse/physician) for stroke?

Yes  (1) No  (2)

1. *If* ***yes,*** *where? ________________________*
2. Have you received any medical rehabilitation? Yes  (1) No  (2)
3. *If* ***yes****, where________________________*
4. *If* ***yes****, what rehabilitation intervention/-s have you received?*

Occupational therapy (1)

Physiotherapy (2)

Language and speech therapy (3)

Other (4)

c)*If* ***other****, what have you received?..........................................................................................*

1. Have you received any assistance from a healer? Yes  (1) No  (2)
2. *If* ***yes****, which assistance did you get?*

Herbal medicine  (1)

Spiritual healing (from religious person)  (2)

Traditional healing (from witchdoctor)  (3)

1. How do you describe the state of your health **now**?

Very good  (1)

Good  (2)

Fair  (3)

Bad  (4)

Very bad  (5)

1. How do you describe the state of your health as compared to your friends of your age **now**? Do you feel it is

Much better  (1)

Little better  (2)

The same  (3)

A little worse  (4)

Much worse  (5)

**Demographics FOR THE FAMILY MEMBERS**

1. **Specify relation to the client** _____________________

*(Example- sister, brother, daughter, son, cousin etc)*

1. **Age:**………………year
2. **Gender**

Male  (1) Female  (2)

1. **Marital status now?**

Married, living together  (1)

Married, not living together  (2)

Unmarried/cohabited  (3)

Single  (4)

Widow/Widower  (5)

Divorced/separated  (6)

1. **Highest level of education**

No education  (1)

Primary school  (2)

Secondary school (O’ level)  (3)

Secondary school (A’ level)  (4)

Tertiary school  (5)

University  (6)

1. **Do you have a source of livelihood now?** Yes  (1) No  (2)

Business  (1)

Salary  (2)

Sale of agricultural products  (3)

Casual labor  (4)

Others  (5)

Specify others............................................................

1. **Staying together with the person with stroke?** Yes  (1) No  (2)
2. **If NO distance from the person with stroke** _____________________________
3. **Who is supporting the patient in her/his home?** ____________________________
