# Supplementary material for: Implementing and Evaluating a Mobile Phone–Supported and Family-Centered Rehabilitation Program for People With Stroke in Uganda (F@ce 2.0): Protocol for a Randomized Controlled Trial
Source: JMIR Res Protoc. 2024 Sep 25;13:e60955. doi: 10.2196/60955 (PMC11464936; doi:10.2196/60955)
Supplement: Multimedia Appendix 4 [file resprot_v13i1e60955_app4.docx]

1. **SELF-EFFICACY SCALE**

The questions below about how confident you are in your ability to perform the following activities. Estimating on a scale from 0 to 10 where 0 is not safe at all and 10 is completely safe. Circle the number that best describes how it usually is.

How confident are you your ability to:

_________________________________________________
 Not sure at all Fully sure

| Go in and out of bed | 1 | 2 | 3 | 4 | 5 | 6 | 7 | 8 | 9 | 10 |
| --- | --- | --- | --- | --- | --- | --- | --- | --- | --- | --- |
| Go to the bathroom | 1 | 2 | 3 | 4 | 5 | 6 | 7 | 8 | 9 | 10 |
| Wash yourself | 1 | 2 | 3 | 4 | 5 | 6 | 7 | 8 | 9 | 10 |
| Dress on/off | 1 | 2 | 3 | 4 | 5 | 6 | 7 | 8 | 9 | 10 |
| Bathing | 1 | 2 | 3 | 4 | 5 | 6 | 7 | 8 | 9 | 10 |
| Walking indoors | 1 | 2 | 3 | 4 | 5 | 6 | 7 | 8 | 9 | 10 |
| Climbing stairs | 1 | 2 | 3 | 4 | 5 | 6 | 7 | 8 | 9 | 10 |
| Take a walk | 1 | 2 | 3 | 4 | 5 | 6 | 7 | 8 | 9 | 10 |
| Cook for yourself | 1 | 2 | 3 | 4 | 5 | 6 | 7 | 8 | 9 | 10 |
| Eat food | 1 | 2 | 3 | 4 | 5 | 6 | 7 | 8 | 9 | 10 |
| Go Shopping | 1 | 2 | 3 | 4 | 5 | 6 | 7 | 8 | 9 | 10 |
| Meet friends and relatives | 1 | 2 | 3 | 4 | 5 | 6 | 7 | 8 | 9 | 10 |
| Using your phone | 1 | 2 | 3 | 4 | 5 | 6 | 7 | 8 | 9 | 10 |
| Be supportive of other people | 1 | 2 | 3 | 4 | 5 | 6 | 7 | 8 | 9 | 10 |
| Make trips to the village | 1 | 2 | 3 | 4 | 5 | 6 | 7 | 8 | 9 | 10 |
| Taxi / Boda boda | 1 | 2 | 3 | 4 | 5 | 6 | 7 | 8 | 9 | 10 |
