# Supplementary material for: Implementing and Evaluating a Mobile Phone–Supported and Family-Centered Rehabilitation Program for People With Stroke in Uganda (F@ce 2.0): Protocol for a Randomized Controlled Trial
Source: JMIR Res Protoc. 2024 Sep 25;13:e60955. doi: 10.2196/60955 (PMC11464936; doi:10.2196/60955)
Supplement: Multimedia Appendix 5 [file resprot_v13i1e60955_app5.pdf]

Beredningsgruppens yttrande

Vetenskapsrådet

2019-03683 Susanne Guidetti **Beredningsgrupp: UF-5**

Utlysningsnamn: Forskningsbidrag Stora utlysningen 2019 (Utvecklingsforskning)

Projekttitel (svenska): Delaktighet i vardagen efter stroke i Uganda - En randomiserad kontrollerad studie av en familjecentrerad intervention som använder mobiltelefonen som stöd i rehabilitering.

Bidragsform: Projektbidrag

Sökt inriktning: Utvecklingsforskning

Scientific quality of the proposed research **6**

1 - Poor, 2 - Weak, 3 - Good, 4 - Very Good, 5 - Very good to excellent, 6 - Excellent, 7 - Outstanding

This is an important, excellent and high-quality study that will add to a scant evidence base on management of stroke in the community in low-income settings. The theoretical foundations are clear and the research questions and methods are both clearly stated and appropriate. Data management methods are clear and appropriate.

The panel raised some minor comments with related suggestions for the work:
(1) the economic cost of the intervention: the team might consider including a study component on cost;
(2) with regard to the process evaluation, it was not very clear what quantitative data will be used. In addition, the team might consider whether developing a theory of change showing how the intervention affects outcomes might be useful as part of the process evaluation
(3) there was a lack of critical reflection on groups not included (non mobile users) and on the long term feasibility of the intervention

Novelty and originality **6**

1 - Poor, 2 - Weak, 3 - Good, 4 - Very Good, 5 - Very good to excellent, 6 - Excellent, 7 - Outstanding

This study appears highly novel, original, and progressing from previous research in the field much of which was done by the study team in Uganda.

Merits of the applicant(s) **6**

1 - Poor, 2 - Weak, 3 - Good, 4 - Very Good, 5 - Very good to excellent, 6 - Excellent, 7 - Outstanding

An impressive team with excellent merits who have conducted a great deal of previous research on the study topic and published it in an efficient way. An enviable track record and a strong scientific network.

Feasibility **3**

1 - Not feasible, 2 - Partly feasible, 3 - Feasible

Overall the project appears feasible. One minor issue to consider: in the randomised trial the comparison group will receive the intervention, according to the ethics statement, and yet the proposal does not elaborate on when this will happen. Although there is a study time plan, this does not show the study from the perspective of the participants -- an additional time plan, from their perspective, would be very useful.

Overall assessment of the application's scientific quality* **6**

1 - Poor, 2 - Weak, 3 - Good, 4 - Very good, 5 - Very good to excellent, 6 - Excellent, 7 - Outstanding

An excellent and scientifically sound study addressing an important and topical issue, proposed by a multi-disciplinary team with a strong track record.

Relevance for call* **3**

1 - Not relevant, 2 - Relevant, 3 - Very relevant

Addressing disability and chronic disease in low income settings are important societal challenges in low income settings, why the study is very relevant for the call.
